# Glutamine Metabolism in Health and Diseases

**DOI:** 10.1002/mco2.71035

**Published:** 2026-09-29

**Authors:** Yi Ding, Fan Tong, Jing Zeng, Chun‐Ping Cui

**Affiliations:** ^1^ State Key Laboratory of Medical Proteomics, Beijing Proteome Research Center, National Center for Protein Sciences (Beijing) Beijing Institute of Lifeomics Beijing China; ^2^ Department of Endocrinology, The Second Medical Center & National Clinical Research Center for Geriatric Diseases Chinese PLA General Hospital Beijing China

**Keywords:** cell metabolism, cellular homeostasis, glutamine, pathological conditions, therapeutic target

## Abstract

Glutamine, the most abundant nonessential amino acid in the blood and tissues, plays essential roles in cellular proliferation, immune regulation, acid–base homeostasis, and metabolic balance. Although traditionally classified as a nonessential amino acid, glutamine becomes conditionally essential under pathological conditions and physiological stress due to its critical role in supporting cellular adaptation. Under diverse pathological states, glutamine metabolism undergoes extensive reprogramming, and cancer cells exhibit a particularly high dependence on glutamine to sustain proliferation, redox balance, and biosynthetic demands. In this review, we summarize the multifaceted functions of glutamine metabolism in physiological and pathological processes. We first discuss the fundamental pathways of glutamine synthesis, transport, and utilization, followed by an overview of its regulatory roles in cellular responses to oxidative, nutritional, thermal, mechanical, DNA damage, and osmotic stresses. We further highlight the involvement of glutamine metabolism in cancer progression, immune regulation, metabolic plasticity, and therapeutic resistance. Finally, we summarize emerging glutamine‐targeted therapeutic strategies, including metabolic inhibitors, combination therapies, and advanced technologies for metabolic imaging and single‐cell analysis. This review provides a comprehensive perspective on glutamine metabolism and highlights its potential as a therapeutic target for cancer and other metabolic disorders.

## Graphical abstract

Glutamine metabolism acts as a metabolic hub connecting cellular stress adaptation, immune regulation, and disease development. Through regulating energy production, biosynthesis, redox balance, and nitrogen metabolism, glutamine supports cellular homeostasis under physiological and pathological conditions. Stress‐induced metabolic reprogramming contributes to cancer progression, immune dysfunction, neurological disorders, and metabolic diseases. Targeting glutamine metabolism through dietary interventions, metabolic inhibitors, and combination therapies provides promising opportunities for future disease treatment.

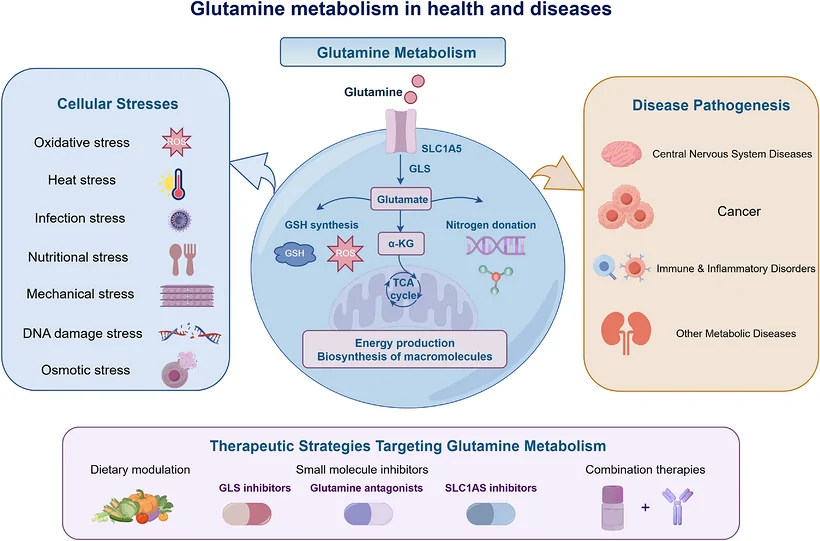

## 1 Introduction

Glutamine is an L‐α‐amino acid containing five carbon atoms and has a molecular weight of 146.15 g/mol. At physiological pH, glutamine is classified as a neutral amino acid. Nutritionally, it is categorized as a nonessential amino acid (NEAA) that is synthesized through the endogenous glutamine biosynthesis pathways [1]. It serves as a crucial precursor for the synthesis of nucleic acids, proteins, neurotransmitters, and various biomolecules [2]. Additionally, glutamine is the most abundant amino acid in the body, with concentrations 10‐ to 100‐fold higher than those of other amino acids in plasma and tissues [2, 3]. It is essential for cellular proliferation, immune function, acid–base homeostasis, and energy metabolism [4]. Furthermore, glutamine plays a significant role in regulating various bodily functions and possesses anti‐inflammatory, antioxidant, and antiapoptotic properties [5]. Glutamine plays an important role in the nitrogen and carbon‐skeleton exchange among different tissues, where this amino acid fulfils many different physiological functions.

Various physiological stressors can cause protein damage and misfolding, ultimately resulting in cellular injury and death. In response, cells have evolved protective mechanisms to enhance survival under adverse conditions. Although most tissues can synthesize glutamine, its demand may exceed endogenous supply during rapid growth or under stress conditions such as major surgery, trauma, and sepsis, rendering it conditionally essential [6, 7]. Glutamine is therefore considered a conditionally essential nutrient during severe injury or illness. Glutamine administration partially reduces inflammation [8, 9] and tissue damage associated with stress [10]. Although glucose is the primary energy source for many cells, glutamine consumption can equal or exceed glucose utilization under catabolic conditions such as malnutrition and oxidative stress [11, 12, 13]. It is also essential for stress‐induced expression of heat shock proteins (HSPs) [14]. In disease contexts, tumor cells exhibit increased glutamine dependence due to rapid proliferation, accompanied by dysregulated expression of glutamine metabolic enzymes and transporters. Through its involvement in energy production, redox regulation, macromolecular synthesis, and signal transduction, glutamine functions as a metabolic fuel supporting multiple biosynthetic pathways in tumor cells [15]. In neurodegenerative diseases, glutamine serves as an important regulator of neurotransmitter synthesis and neuronal energy metabolism. Disruption of glutamine metabolic homeostasis may contribute to disease initiation and progression [16, 17].

Given the significance of glutamine metabolism in cellular stress responses, understanding its functions is essential for developing therapeutic strategies. This review comprehensively explores the multifaceted roles of glutamine metabolism under physiological and pathological conditions. First, the basic metabolic pathways of glutamine will be elaborated in detail. Second, the critical roles of glutamine metabolism in maintaining normal physiological functions of the organism will be thoroughly analyzed, with a particular focus on the abnormal changes in glutamine metabolism and its underlying mechanisms during stress responses and the development and progression of various diseases. Additionally, the therapeutic potential of targeting glutamine metabolism is discussed. The feasibility of developing novel glutamine‐targeted therapeutic strategies is evaluated.

## 2 Physiological Roles of Glutamine in Health

Glutamine is transported into cells through plasma membrane glutamine transporters, including solute carrier family 1 member 5 (SLC1A5), SLC38A1, and SLC38A2 [18]. For mitochondrial glutaminolysis, cytosolic glutamine must be transported across the inner mitochondrial membrane by the mitochondrial isoform of SLC1A5 [19]. The catabolism of glutamine starts with its transformation into glutamate, a process that either contributes the amide nitrogen to biosynthetic pathways or releases it as ammonia. This reaction is catalyzed by glutaminases (GLSs), including isoenzymes encoded by the *GLS* and *GLS2* genes [20, 21, 22]. These isozymes are localized in the mitochondria. Mitochondrial glutamate generated through these catabolic pathways can be exported to the cytosol via the SLC25A18 and SLC25A22 transporters [23]. The nitrogen from glutamine plays a crucial role in sustaining the various amino acid pools within the cell, primarily through the action of aminotransferases. Beyond transamination reactions, glutamate‐derived carbon and nitrogen contribute to proline synthesis, which is essential for collagen production [24]. Macrophages utilize glutamate to synthesize arginine, which is subsequently converted into nitric oxide (NO) through the action of the inducible NO synthase (iNOS) enzyme, using NADPH as a cofactor [25]. In the cytosol, glutamate contributes to glutathione (GSH) and nonessential amino acid (NEAA) biosynthesis and facilitates extracellular cystine uptake through SLC7A11 [1]. Mitochondrial glutamate is subsequently converted into α‐ketoglutarate (α‐KG) by glutamate dehydrogenase 1 (GLUD1/GDH1) or mitochondrial transaminases, including glutamate‐pyruvate transaminase 2 (GPT2) and glutamate‐oxaloacetate transaminase 2 (GOT2). A portion of α‐KG is exported from mitochondria to the cytoplasm via SLC25A11 to support fatty acid biosynthesis and NADH production, whereas the remaining mitochondrial α‐KG enters the tricarboxylic acid (TCA) cycle, feeding into either oxidative phosphorylation (OXPHOS) or reductive carboxylation [26]. α‐KG is reductively carboxylated by NADPH‐dependent isoforms of isocitrate dehydrogenase to produce isocitrate, citrate, and other TCA cycle intermediates [15]. Citrate is especially crucial for lipid synthesis under hypoxic conditions [27, 28]. Additionally, in the oxidative phosphorylation pathway, glutamine metabolites play a crucial role in generating electron donors such as NADH and flavin adenine dinucleotide, reduced (FADH_2_). These electron donors are specifically used in the mitochondrial electron transport chain for the synthesis of adenosine triphosphate (ATP) [29]. Moreover, GTP is produced in the Krebs cycle, where NADH and FADH2 are also generated (Figure 1). In particular, glutamine is crucial in this process as it supplies carbon to the TCA cycle and serves as a nitrogen source for synthesizing alanine, aspartate, and serine [30].

**FIGURE 1:**
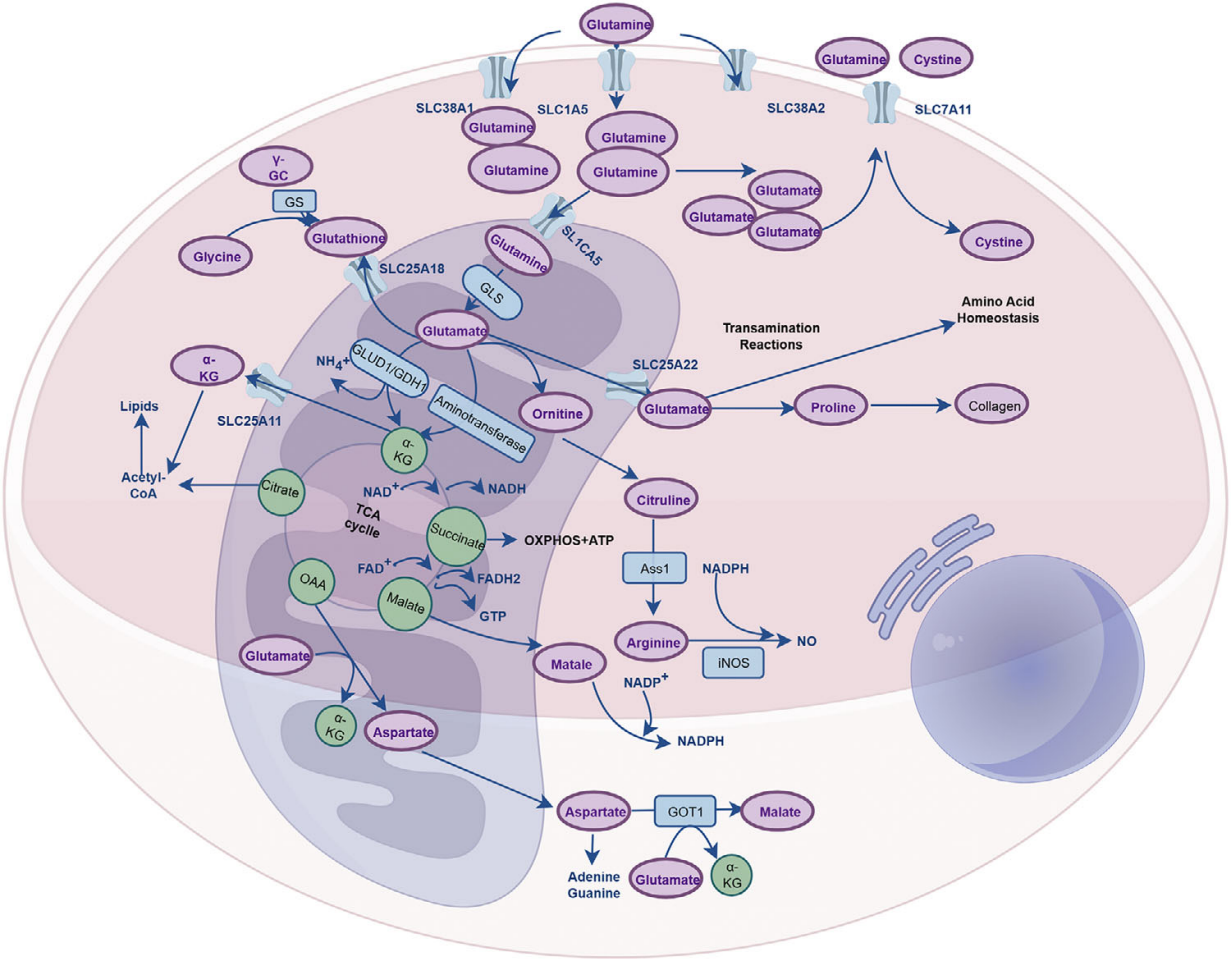
Major metabolic and biosynthetic pathways of glutamine. Glutamine is transported into cells through plasma membrane glutamine transporters, such as SLC1A5, SLC38A1, and SLC38A2, and transported out by SLC7A11. Glutamine could be converted to glutamate and then, with glycine, contribute to the synthesis of glutathione by glutamate–cysteine ligase in the cytosol. Glutamine must be transported into the mitochondria by the SLC1A5 variant. Once inside, it is converted into glutamate through the action of the enzyme glutaminase (GLS or GLS2). Subsequently, glutamate is transformed into α‐ketoglutarate (αKG) via one of two groups of enzymes: either glutamate dehydrogenase (GLUD1 or GLUD2, collectively known as GLUD) or aminotransferases. α‐ketoglutarate enters the tricarboxylic acid (TCA) cycle, generating energy for cellular functions. Malate, which exits the TCA cycle, can be converted into pyruvate and NADPH, thus providing essential reducing equivalents. Additionally, citrate can be transformed into acetyl‐CoA to engage in lipid metabolism. Furthermore, oxaloacetate (OAA) can be converted into aspartate, thereby contributing to the synthesis of various amino acids. Glutamate may also be involved in the metabolism of arginase and nitric oxide, mediated by the actions of arginosuccinate synthetase (ASS1) and nitric oxide synthase (NOS).

### 2.1 Transporters and Enzymes

SLC1A5 (also known as ASCT2), an obligatory sodium‐dependent transporter for neutral amino acids [31]. Despite its original name, AlaSerCys Transporter 2 (ASCT2), glutamine is the key amino acid that underpins the unique roles of this transporter in human physiology and pathology [32]. It contains two transcription initiation sites that produce a long transcript encoding the plasma membrane transporter and a shorter variant (SLC1A5_var) that encodes the mitochondrial glutamine carrier [19, 31]. The SLC1A5 structure is characterized by a homotrimer that adopts an outward‐facing conformation with a GltPH‐like fold [33]. The transport mechanism of SLC1A5 resembles an elevator, with hairpin 2 serving as the doors to transport glutamine [34, 35]. The C467 residue in SLC1A5 is essential for both substrate binding and the modulation of transport activity. The lack of a thiol group at this position disrupts hydrogen bond formation, resulting in a unidirectional transport mechanism. Additionally, the transport activity is affected by reducing or oxidizing agents, which correlate with the reduced (thiol) or oxidized (disulfide) states of the cysteine residues in the protein. Consequently, the interconversion between the disulfide and thiol forms of these cysteine residues can toggle SLC1A5's transport function from an “OFF” state to an “ON” state [36, 37]. Consequently, it influences the transport of glutamine into cells. SLC1A5 is widely expressed and plays a key role in distributing glutamine to various tissues [38]. This is crucial for maintaining nitrogen balance, especially in nervous tissue [39]. In addition, SLC1A5 is associated with mTOR signaling, which is especially responsive to intracellular levels of glutamine and asparagine [40]. Several findings show that silencing the ASCT2 gene in hepatoma cells leads to mTOR activation, which represses cell growth and induces apoptosis [40, 41]. Consequently, SLC1A5 has emerged as a promising therapeutic target in oncology [42, 43]. In addition to cancer, altered ASCT2/SLC1A5 activity has also been implicated in neurological disorders, including amyotrophic lateral sclerosis and Parkinson's disease [44, 45].

GLS expression and activity have been found in various human tissues, including the kidneys, liver, brain (mainly in neurons), pancreas, airway epithelium, cardiac muscle, skeletal muscle, small intestine, platelets, and fibroblasts. GLS1, also known as kidney‐type glutaminase, is primarily expressed in the kidneys and brain [46, 47]. At the cellular level, GLS is most likely located in the mitochondria [48]. In some cancers, GLS2 is regarded as a tumor suppressor, with its overexpression linked to reduced overall survival rates [49]. Conversely, recent studies have indicated that GLS1 is often overexpressed in multiple cancer types [50]. The human GLS2 gene has been recognized as a target of p53, meaning that the activation of p53 leads to the expression of GLS2 mRNA [51]. Consequently, GLS2 may play a role in tumor suppression by mediating some of the effects associated with p53^39^. Therefore, different glutaminases play diverse roles in various biological processes and may be involved in the development and progression of different types of cancer [52].

Two highly homologous human genes, *GLUD1* and *GLUD2*, encode two glutamate dehydrogenase isoenzymes. *GLUD1* is located on chromosome 10q and encodes the isoenzyme glutamate dehydrogenase‐1 (GDH1). *GLUD2* is an intronless gene mapped to the Xq chromosome that encodes the glutamate dehydrogenase‐2 isoenzyme (GDH2) [53]. GDH catalyzes the reversible oxidative deamination of glutamate to produce α‐ketoglutarate and a free ammonium ion [39]. Under normal conditions, GDH is a key regulator of amino acid and ammonia metabolism in the human pancreas, liver, and brain [54]. Dysfunction of GDH may contribute to the onset of various neurodegenerative diseases, including Parkinson's disease, epilepsy, Alzheimer's disease, schizophrenia, and frontotemporal dementia [55]. Moreover, GDH is critical for the survival of glioblastoma cells facing impaired glucose metabolism or disrupted Akt signaling. The study revealed that, although cells can still utilize glucose when GDH activity is diminished, they become fully reliant on GDH for growth when glucose is absent or when glucose metabolism is compromised [56]. Increased GDH activity detected in tumors has been identified as a potential prognostic marker for colorectal cancer patients and may also serve as an indicator of metastasis [57]. In addition, gain‐of‐function mutations in the GLUD1 gene enhance GDH1 activity by making the isoenzyme resistant to allosteric inhibition by GTP, while still allowing ADP and L‐leucine to stimulate its action. This results in constant enzyme overactivity, which can lead to hyperinsulinism‐hyperammonemia syndrome [53, 58].

Tissues including the lungs, liver, brain, skeletal muscle, and adipose tissue exhibit tissue‐specific glutamine synthetase activity [2]. The tissues demonstrate reduced glutamine synthetase under certain conditions, including decreased carbohydrate [59] and amino acid intake [60], elevated catabolic states, and the presence of various diseases or stressors [61]. Importantly, the metabolic rewiring of glutamine metabolism is a recognized hallmark of cancer, as it not only supplies substrates for the rapid proliferation of tumor cells but also plays a crucial role in facilitating tumor immune escape.

### 2.2 Systemic Homeostasis

Systemic homeostasis is fundamentally orchestrated by glutamine, which acts as a central integrative hub that aligns cellular metabolism with whole‐body physiological demands. Under physiological conditions, glutamine sustains cellular homeostasis via three core metabolic functions. First, it serves as a critical nitrogen source for the synthesis of nucleotides, proteins, and nonessential amino acids [62]. Second, it is metabolized to α‐KG, which enters the TCA cycle to support basal energy production [15]. Third, it modulates redox balance by providing precursors for GSH synthesis [63]. Critically, these intracellular functions are embedded within a systemic interorgan circuit that spans intestinal uptake, hepatic interconversion, muscular mobilization, and renal recycling, and integrates with glycolytic and fatty acid oxidation fluxes [1]. This network continuously recalibrates nitrogen, carbon, and redox fluxes in response to fluctuating metabolic states, matching peripheral demands with central supply and thereby epitomizing systemic homeostasis.

### 2.3 Organ‐Specific Functions

Glutamine functions as the body's primary systemic nitrogen carrier, driving interorgan nitrogen flux that connects the metabolic activities of skeletal muscle, liver, intestine, and immune cells [2]. Glutamine contains two nitrogen atoms: the γ‐nitrogen and the α‐nitrogen [64]. The γ‐nitrogen is predominantly utilized in nascent nucleotide biosynthesis, whereas the α‐nitrogen primarily fuels amino acid synthesis. Skeletal muscle serves as the principal site for de novo glutamine synthesis mediated by glutamine synthetase [65]; once released into the circulation, glutamine is transported to visceral organs. Under physiological conditions, the small intestine and liver are its major consumers [66]. Beyond nitrogen transport, glutamine is the primary substrate for renal ammoniagenesis, a process directly coupled to systemic acid–base homeostasis. In the proximal tubule, the catabolism of each glutamine molecule generates two NH_4_^+^ and two HCO_3_^−^ ions: the former is excreted into the urine to eliminate protons, whereas the latter is returned to the bloodstream to replenish the alkali reserve [67]. During metabolic acidosis, renal glutamine extraction increases markedly, driven by upregulated expression of glutaminase, glutamate dehydrogenase, and phosphoenolpyruvate carboxykinase [68].

In the gut, glutamine maintains mucosal barrier integrity by promoting enterocyte proliferation, regulating tight junctions, and suppressing pro‐inflammatory signaling [69]. In the immune system, glutamine fuels lymphocyte proliferation, macrophage phagocytosis, and neutrophil function while also promoting M2 macrophage polarization [2, 70, 71]; beyond these effector roles, it serves as a bioenergetic substrate for leukocytes and critically supports tissue repair and the intracellular signaling cascades involved in pathogen recognition [72].

## 3 Glutamate–Glutamine Cycle in the CNS

### 3.1 Astrocyte–Neuron Metabolic Coupling

The precise regulation of synaptic neurotransmission is crucial for normal brain function, relying on tightly coordinated crosstalk between neurons and astrocytes [73]. During synaptic transmission, presynaptic neurons release glutamate and γ‐aminobutyric acid (GABA) into the synaptic cleft, enabling their binding to postsynaptic receptors to mediate neural signal conduction. Efficient clearance of synaptic glutamate and GABA is indispensable for high‐fidelity rapid signal transmission; in the case of glutamate, this clearance process further prevents harmful, excessive excitatory activation—a phenomenon termed excitotoxicity [74]. Through cooperative interactions, neurons and astrocytes ensure, they ensure a stable supply of excitatory neurotransmitters while preventing neurotoxic damage, which is essential for maintaining neural signaling and brain homeostasis. Furthermore, functional defects in neurons and glial cells are closely linked to the pathogenesis of various neurodegenerative diseases.

### 3.2 Role in Neurotransmission and Energy Metabolism

In neurons, glutamate is repackaged and stored in synaptic vesicles through specific vesicular glutamate transporters [75]. Upon the arrival of an action potential, stored glutamate is released from presynaptic terminals, glutamate can be released upon the arrival of an action potential. This release induces the opening of voltage‐dependent calcium channels, increasing the intracellular calcium (Ca^2^^+^) concentration. The influx of Ca^2^^+^ promotes vesicle fusion with the plasma membrane, resulting in the release of glutamate into the synaptic cleft of the tripartite synapse [76]. Glutamate taken up by astrocytes is converted into the nonneurotoxic amino acid glutamine through a reaction catalyzed by glutamine synthetase [77]. Subsequently, glutamine is released into the extracellular space via sodium‐coupled neutral amino acid transporters and transported back into neurons through specific glutamine transporters [78]. The glutamate–glutamine cycle is tightly coupled to cellular energy metabolism. Astrocytic TCA cycle activity is essential for sustaining this neurotransmitter recycling process [79]. Astrocytes exhibit distinctive metabolic characteristics, including the coordinated glycogen metabolism and pyruvate carboxylation, which are essential processes supporting high levels of glutamine synthesis and export [80, 81].

### 3.3 Dysfunction in Brain Disorders

Dysregulation of the glutamate–glutamine cycle is a common feature of various neurological disorders. This dysfunction may be associated with impaired glutamate uptake, disruption of glutamine homeostasis, or broader disturbances in cellular metabolism. Alterations of glutamate or glutamine metabolism are implicated in the pathogenesis of Alzheimer's disease (AD). In both AD patients and mouse models, reduced glutamate release from presynaptic terminals in the brain contributes to impaired synaptic transmission between glutamatergic neurons [82]. For instance, glutamine synthetase activity [83], as well as the conversion and export of glutamine from astrocytes, are decreased in AD patients and animal models [84]. Studies have demonstrated that ceftriaxone treatment not only enhances glutamate uptake but also promotes glutamine synthesis, transport, and metabolism in mouse models of AD [82, 83]. Reduced TCA cycle activity in astrocytes may impair glutamate oxidative metabolism. Coupled with compromised glutamine synthesis, this disruption can lead to intracellular glutamate accumulation, thereby reducing the glutamate uptake capacity of astrocytes [74]. The consequences of glutamate–glutamine cycle dysfunction extend beyond AD; similar perturbations have been implicated in epilepsy. Epileptic seizures are primarily caused by the relative imbalance between excitatory and inhibitory neurotransmitters, including glutamate and GABA, leading to abnormal and excessive neuronal synchronization in the brain [85].

Impaired glutamine synthesis reduces inhibitory neurotransmission and promotes neuronal hyperexcitability, potentially contributing to epileptogenesis. Paradoxically, oral glutamine supplementation not only has failed to provide therapeutic benefits in established epileptic models but also may even aggravate seizure activity [86].

## 4 Glutamine in Cellular Stress and Adaptation

As the most abundant free amino acid in the human body, glutamine plays a critical role in maintaining cellular homeostasis and facilitating adaptation to stress conditions, such as oxidative stress, nutrient deprivation, and inflammatory responses.

### 4.1 Oxidative Stress

Oxidative stress, characterized by an imbalance between oxidant production (primarily reactive oxygen species, ROS) and antioxidant defense capacity, has been implicated in neurodegenerative diseases, intestinal disorders, and various other pathological conditions (Figure 2) [87]. Excessive ROS accumulation during oxidative stress can cause severe damage to cellular macromolecules. Glutathione (GSH), a tripeptide composed of glutamate, cysteine, and glycine (Glu‐Cys‐Gly), serves as a major endogenous antioxidant and plays a pivotal role in scavenging peroxide radicals [88]. As glutamine is a precursor to GSH, supplementing it in clinical diets can help maintain high GSH levels and protect against oxidative stress‐induced damage [63]. Glutamine has been reported to alleviate bronchopulmonary dysplasia by reducing pulmonary inflammation, oxidative stress, and apoptosis while improving lung function [89]. Glutamine deprivation reduced GSH production; oral supplementation with L‐glutamine plus L‐alanine or L‐alanyl‐L‐glutamine dipeptide (DIP) is effective in attenuating oxidative stress and inflammation induced by endotoxemia in mice [5]. Simultaneously, the prolonged oral intake of DIP and free L‐glutamine plus L‐alanine prior to extended exercise serves as an efficient source of glutamine and glutamate, potentially increasing muscle and liver GSH levels and enhancing the redox state of the cells [90]. Also in the liver, L‐glutamine plus L‐alanine administration alleviates lipopolysaccharide (LPS)/D‐galactosamine‐induced hepatic apoptosis and significantly reduces cleaved caspase‐3 protein levels [91]. Pretreatment with glutamine reduces oxidative damage in the liver [92]. Eder et al. demonstrated that supplementation with L‐glutamine plus L‐alanine or DIP restored plasma and muscle glutamine levels, improved GSH balance and redox status in erythrocytes and skeletal muscle, increased muscle HSP70 expression, reduced oxidative stress, and suppressed tumor necrosis factor‐α (TNF‐α)‐mediated nuclear factor kappa‐B (NF‐κB) pathway activation [93]. Administering glutamine to sodium sulfate salt‐induced colitis mice significantly reduced oxidative stress‐induced injury and inhibited the activity of the phosphatidylinositol‐3‐kinase/protein kinase B (PI3K/Akt) signaling pathway [94]. Takehiro et al. reported that glutamine ensures ROS‐dependent spermatogonial stem cell self‐renewal by providing protection against NADPH oxidase 1 (NOX1) and inducing Myc [95]. Under inflammatory or hypoxic conditions, glutamine treatment enhanced tubular epithelial cell viability and reduced kidney damage in vivo during acute kidney injury [96]. Furthermore, combined strength and endurance exercise with L‐glutamine supplementation reduces intestinal inflammation and jejunal damage while improving cytokine profiles and oxidative stress parameters [97, 98]. Consistently, glutamine can improve intestinal Ca^2+^ absorption altered by oxidative stress [99]. Glutamine's regulation of xanthine oxidase, uric acid, and lactate may help alleviate intestinal injury and dysmotility induced by ischemia‐reperfusion [100].

**FIGURE 2:**
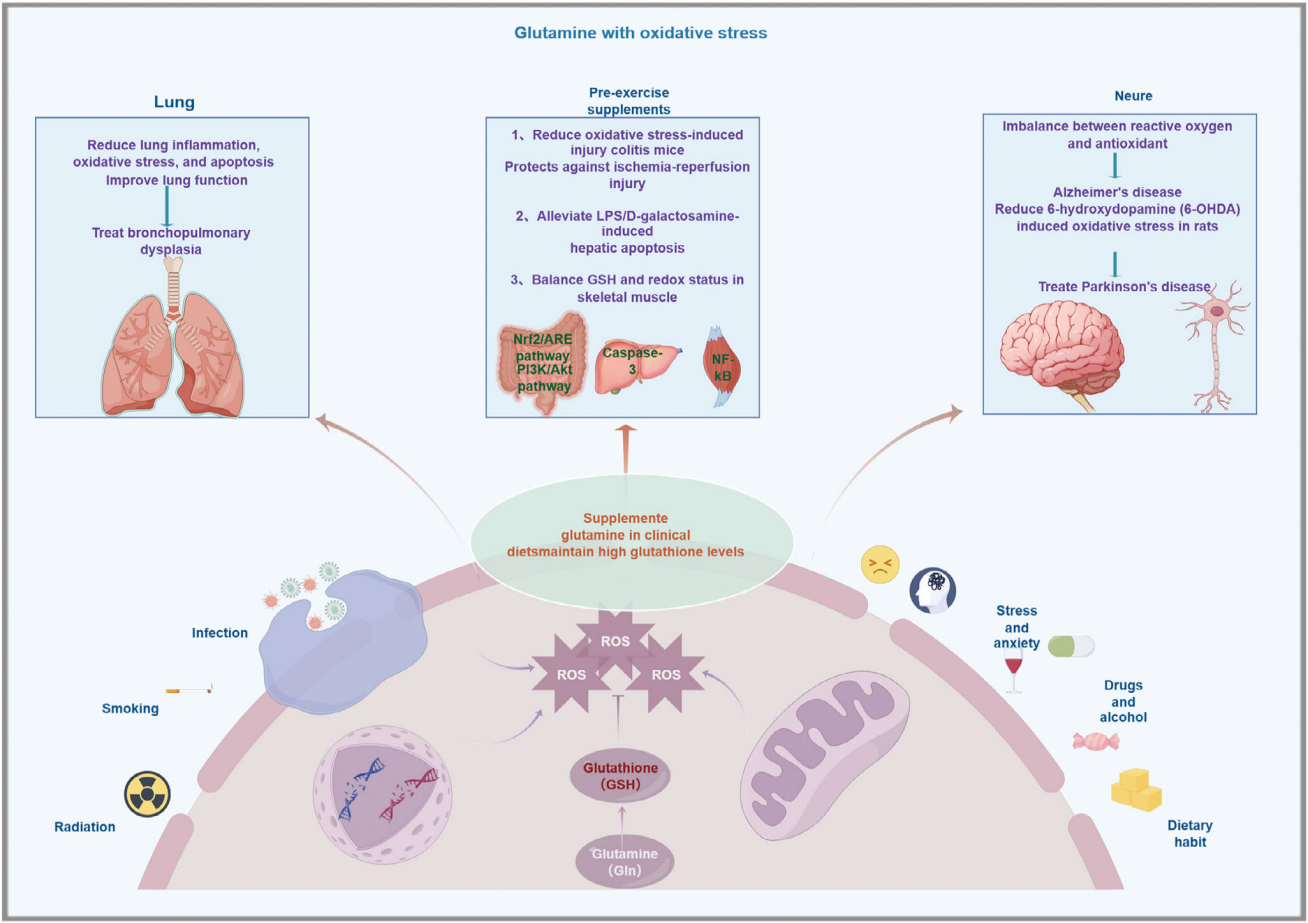
Glutamine and oxidative stress. The cartoon depicts the effects of oxidative stress on different organs. ROS can be produced through multiple pathways such as radiation, smoking, infection, stress and anxiety, drugs and alcohol use, dietary habits, etc. When ROS levels become excessive, it leads to oxidative stress. Glutathione (GSH), the metabolite of glutamine, can directly inhibit ROS levels and neutralize free radicals. Glutamate metabolism plays an important role in intracellular ROS homeostasis. Supplementing glutamine in clinical diets maintains high GSH levels. Targeting glutamine metabolism disrupts the oxidative balance.

An imbalance between ROS and antioxidant defenses often triggers neuronal damage and death [101]. Changes in glutamine metabolism seem to play a particularly important role in neurodegenerative diseases [102]. The overexpression of the SLC1A5 variant significantly ameliorates motor symptoms in Parkinson's disease and rescues dopaminergic neuron loss in the midbrain and striatum, indicating its potential therapeutic efficacy for this disease [103]. Short‐chain fatty acids alleviate the deficit in glutamate delivery within astrocyte–neuron coupling by modulating glutamine synthetase, thereby safeguarding neurons from oxidative damage [104]. It may serve as a valuable adjunctive therapy for treating. In summary, glutamine protects against oxidative stress primarily by serving as a precursor for GSH synthesis. Glutamine supplementation, either alone or combined with alanine or alanyl‐glutamine dipeptide, alleviates oxidative stress‐associated tissue injury in the lung, liver, intestine, kidney, and nervous system through regulation of pathways including PI3K/Akt and Nrf2/ARE signaling.

### 4.2 Heat Stress

The heat shock response is a fundamental cellular defense mechanism that enables organisms to maintain proteostasis and survive under stressful conditions. Several studies have demonstrated that glutamine acts as a potent enhancer of the heat shock response [105, 106, 107, 108]. This protective mechanism is primarily mediated by a highly conserved family of proteins known as heat shock proteins (HSPs). Glutamine‐induced upregulation of HSP expression has been suggested to contribute significantly to the protection of cells, tissues, and whole organisms against stress‐induced injury [109, 110].

Early studies indicated that under heat stress, HSP levels were up to 100 times higher with glutamine compared with glutamate alone [111]. Activation of HSP signaling pathways has been implicated in the protective effects of glutamine at cellular, tissue, and organismal levels [112, 113]. Glutamine safeguards intestinal cells from heat injury and provides protection following heat stress by activating the PI3‐K/Akt signaling pathway, which prevents fibronectin‐integrin expression and promotes an increase in HSP70 expression [10, 114]. Furthermore, glutamine supplementation can mitigate lethal heat injury and enhance HSP72 expression in intestinal epithelial cells. The researchers discovered that glutamine exhibited maximal protective effects at concentrations close to 8 mM [115]. In addition, glutamine depletion increased cell apoptosis after heat stress, which was inhibited by treatment with inhibitors of mTOR and p38 MAP kinase [116]. Glutamine supplementation also protects chondrocytes; glutamine protected the cells from heat stress and NO‐induced apoptosis [117]. Glutamine is required for the induction of HSP70 and HSP25 during heat stress, whereas supplementation with ornithine or polyamines restores heat‐induced expression of these proteins [118]. Moreover, glutamine specifically increases HSP72 transcript abundance and enhances HSF‐1 DNA‐binding activity during heat shock [119]. Following LPS exposure, glutamine may inhibit monocyte and lymphocyte HSP72 expression and subsequently enhance it after heat shock induction; however, it may not influence the early induction of HSP72 mRNA [120]. Oral glutamine supplementation increases tissue HSP70 expression and HSF‐1 activation after hyperthermia, suggesting potential benefits in preventing heatstroke‐associated mortality in vulnerable populations [114]. Glutamine also enhances lung HSP70 and HSP25 expression and improves survival after cecal ligation and puncture‐induced sepsis [113]. Physiological levels of glutamine appear inadequate for maximizing HSP expression following environmental stress or injury, particularly during critical illness, trauma, burn injuries, or major surgeries, which can significantly reduce plasma glutamine levels [121]. A key mechanism by which glutamine enhances outcomes following illness and injury may be its modulation of the HSP pathway, indicating its potential role as a “drug” rather than just a nutrient. In summary, glutamine enhances the heat shock response by promoting HSP expression through pathways such as PI3K/Akt signaling, thereby protecting cells against heat‐induced apoptosis and improving survival outcomes in models of sepsis and heatstroke. These findings highlight the therapeutic potential of glutamine beyond its conventional nutritional functions.

### 4.3 Infection Stress

Severe infections disrupt glutamine distribution among multiple organs, leading to substantial alterations in tissue‐specific glutamine transport and intracellular glutamine availability [122]. Based on the type of pathogen involved, infections can be classified as viral, bacterial, fungal, or parasitic. Increasing evidence indicates that diverse infectious stimuli induce metabolic reprogramming in immune cells, which is essential for mounting effective host responses against microbial pathogens [123, 124]. As the most abundant free amino acid in the human body, glutamine serves as a critical metabolic substrate and energy source for various cell types [125]. Its importance in infection‐associated stress responses is increasingly recognized; however, the effects of glutamine metabolism vary depending on the pathogen and the host immune context. Viruses, as obligate intracellular parasites, depend on host metabolic pathways to obtain energy and biosynthetic precursors required for replication and infection. Viral infection induces metabolic reprogramming in host cells, thereby influencing disease progression and infection outcomes [126]. The specific details are as follows: as the primary carbon source for the TCA cycle, infection increases the uptake of glutamine in cells [127, 128, 129, 130, 131, 132, 133] and elevates the metabolism of glutamine in cells [127, 133, 134, 135]. Glutaminolysis is also essential for virus replication [128, 131, 136, 137, 138, 139, 140, 141, 142] and optimal Kaposi's Sarcoma production [139]. During bacterial infection, immune cells consume glutamine at rates comparable to or exceeding those of glucose, highlighting the importance of glutamine metabolism in antibacterial immune responses [143, 144]. The catabolism of glutamine is crucial for the M1‐like polarization of macrophages during *Mycobacterium tuberculosis* infection [145]. Adi Haber discovered that the intracellular concentration of L‐glutamine in cells functions as an on/off switch for virulence gene induction. Specifically, the induction of these genes only takes place once the L‐glutamine levels within Listeria reach a specific threshold [146]. A study discovered that glutamine metabolism is significantly upregulated in macrophages infected with *Aspergillus fumigatus*, and that depletion of glutamine leads to a reduction in the antifungal effector functions of these macrophages [147]. Furthermore, innate immune responses against *Candida albicans* require activation of glutaminolysis [148]. Given the critical roles of glutamine in the infection process, understanding its effects on the immunometabolic properties of various pathogenic bacteria could significantly aid in the development of novel therapeutic strategies for managing infections (Table 1). Elucidating the mechanisms by which glutamine regulates cellular metabolism and immune function during infection may reveal potential targets to enhance host defense or modulate pathogen‐associated metabolic processes.

**TABLE 1:** The interplay between pathogen infections and glutamine metabolism.

Pathogenic bacteria name	Effect on glutamine metabolism	Reference
Poliovirus	Glutamine significantly enhanced the maximum yields of poliovirus.	[128, 138]
Human cytomegalovirus (HCMV)	Glutamine uptake increases.	[129]
Vaccinia virus (VACV)	Deprivation of external glutamine in Vaccinia virus (VACV)‐infected cells led to a substantial reduction in the yield of infectious virus particles.	[149]
Herpes simplex virus (HSV)	Inhibition of glutamine metabolism altered the degree of reactivation from latency and delayed the time of viral replication.	[141]
Nairovirus	The plasma glutamine levels were found to be decreased in the patient cohort.	[150]
Hepatitis C virus (HCV)	The metabolism of glutamine was observed to be upregulated in cells infected with HCV, and it has been demonstrated to be essential for HCV replication.	[131]
Adenovirus	Adenovirus enhances glutamine uptake and metabolism, facilitating the generation of optimal progeny virions.	[132]
Human immunodeficiency virus (HIV)	Glutamine is a major energy source of HIV infected macrophages.	[135]
Noroviruses (NoVs)	During MNV‐1 infection of macrophages, glutaminolysis is significantly upregulated. Glutamine deprivation adversely impacts the MNV lifecycle at the genome replication stage.	[142]
Human immunodeficiency virus type 1 (HIV‐1)	Glutamine concentrations are elevated in HIV‐1‐infected cells, accompanied by changes in cellular glutamine metabolism.	[133]
African swine fever virus (ASFV)	ASFV infection promotes the utilization of glutamine as a metabolic substrate to enhance self‐replication.	[140]
Kaposi's sarcoma‐associated herpesvirus (KSHV)	KSHV infection of endothelial cells enhances host cell glutamine metabolism. KSHV infection results in increased glutamine uptake and elevated levels of intracellular glutamine. Glutaminolysis is crucial for early gene translation and is necessary for optimal KSHV virion production.	[130, 139]
Rubella virus (RV)	The requirement of rubella virus for exogenous glutamine to support efficient replication.	[137]
Human papillomavirus type 16 (HPV‐16)	Elevated glutamine consumption and enhanced glutaminolysis are observed in cells expressing the E7 oncoprotein of HPV‐16.	[134]
Rhinovirus (RV)	Glutamine deprivation led to compromised replication of RV.	[136]
Human respiratory syncytial virus (HRSV)	Elevated levels of glutamine and glutamic acid are observed in infected cells.	[127]

### 4.4 Nutritional Stress

Nutritional stress disrupts the cellular redox homeostasis, leading to an increased production of ROS [151]. Under glucose deprivation, glutamine‐derived metabolites, including fumarate, malate, and citrate, are significantly increased [152]. Recent studies have demonstrated that lactate and glutamine promote NADPH generation through isocitrate dehydrogenase 1 (IDH1) and malic enzyme 1 (ME1), respectively, in conditions of glucose deprivation [153]. Additionally, glutamine serves as an energy source for cultured neurons when glucose availability is limited [154]. When astrocytes at different developmental stages were cultured in the presence or absence of glucose, glucose deprivation reduced the specific activity of glutamine synthetase [155]. Moreover, both glutamine and pyruvate (at 2 mM) preserve mitochondrial membrane potential and reduce cell death in cerebellar granule neurons during glucose deprivation [156].

Tumor cells undergo metabolic reprogramming to adapt to nutrient stress, with glutamine recognized as one of the most important metabolic substrates after glucose [157, 158]. In tumors, inhibition of amino acid uptake selectively reduces the viability of glutamine‐deprived, autophagy‐deficient cells, but has minimal effects on wild‐type cells. This suggests that autophagy‐deficient tumor cells are reliant on the uptake of extracellular amino acids [159]. In addition, atorvastatin can prevent cell death induced by oxygen‐glucose deprivation, potentially through regulation of glutamate uptake and glutamine synthetase activity, along with a reduction in oxidative stress [11]. GLUD1‐mediated glutaminolysis promotes the survival of hepatocellular carcinoma cells during glucose deprivation [160]. In colorectal cancer cells, EZH2 deficiency increases GLS expression of GLS, enhancing the production of glutamate. Elevated intracellular glutamate subsequently increases GSH levels, which ultimately reduces the cell death caused by ROS during conditions of glucose deprivation [161]. Recent studies have shown that the depletion of both glucose and glutamine leads to metabolic reprogramming and growth inhibition, highlighting a potential therapeutic strategy based on targeting cancer cell metabolism [19, 162, 163].

### 4.5 Other Cellular Stress

#### 4.5.1 Mechanical Stress

Multicellular organisms expose their constituent cells to ongoing mechanical stress. Cellular protective systems resisting such mechanical insults govern cell differentiation, adhesion, and migration [164]. Tissue transglutaminase plays a role in the osteogenic differentiation of human ligamentum flavum cells induced by mechanical load [165]. Recently, it has been observed that inflammation, hypoxia, and mechanical stress activate the transcriptional coactivators YAP and TAZ in pulmonary arterial adventitial fibroblasts. This activation leads to increased glutamine and serine catabolism, supporting proline and glycine production and enhancing collagen synthesis. Interventions targeting proline and glycine metabolism can reduce vascular stiffness and improve cardiovascular function in pulmonary hypertension rodent models [166]. Mechanical signals from the tumor microenvironment affect cell mechanics and alter glutamine metabolism in cells, thereby enhancing cancer aggressiveness [167]. These studies have significant implications for our fundamental understanding of the connection between mechanobiology and metabolism in both health and disease, offering new therapeutic opportunities for intervention strategies.

#### 4.5.2 DNA Damage Stress

The preservation and integrality of genomic sequence information in living organisms is crucial for the continuity of life. Concurrently, mutagenesis plays an essential role in both the maintenance and evolution of genetic material. While it fosters diversity and adaptation, it also contributes to the development of cancer, various human diseases, and the aging process. DNA damage can be classified into two primary categories based on its origin. The first category is endogenous DNA damage, which encompasses the following types: replication errors, DNA base mismatches, topoisomerase‐DNA complexes, spontaneous base deamination, a basic site, and abnormal DNA methylation. The second category is exogenous DNA damage, which includes ionizing radiation (IR), ultraviolet (UV) radiation, and various external chemical agents [168]. Subsequently, Haulica found that whole‐body exposure to X‐rays resulted in a significant activation of glutaminase, which contributed to an increase in free ammonia concentration in the rat brain [169]. Yang et al. [171] found that IR enhanced glutamine metabolism in tumor cells [170]. Mass spectrometry‐based metabolite profiling revealed that the level of glutamine in mouse liver increased following exposure to ultraviolet B (UVB) radiation. After exposure to silica, the level of glutamine increases in human alveolar type II epithelial cells (A549 cells). Furthermore, supplementing glutamine into the cell culture can significantly boost the expression levels of both EMT‐related markers and Snail (a zinc finger transcription factor) [172].

#### 4.5.3 Osmotic Stress

Under physiological conditions, most mammalian cells are rarely exposed to substantial osmotic fluctuations. The osmolality of bodily fluids generally remains at 285 mOsmol/kgH_2_O and is tightly regulated within a narrow range through body fluid homeostasis mechanisms [173]. In addition to maintaining a stable cell volume during resting states, most studied cells can also counteract volume fluctuations through adaptive mechanisms, including regulatory volume decrease (RVD) and regulatory volume increase (RVI). In maintaining cellular osmotic balance, glutamine also plays an important role. For example, Valeria Dall'Asta found that under hypertonic conditions, the most significant change observed during RVI was that the intracellular L‐glutamine concentration increased by greater than 70% in the incubated human fibroblasts [174]. María C. Hyzinski‐García found that in astrocytes, a 40% reduction in medium osmolarity moderately stimulated the release of L‐glutamine by approximately twofold, while having no effect on uptake of L‐glutamine. Additionally, cellular swelling inhibits astrocytic glutamine synthetase activity. These combined effects are likely to reduce the export of glutamine from astrocytes in vivo, which may partially account for the hyperexcitability and seizures observed in cases of human hyponatremia [175]. Adequate volume regulation is crucial for cellular processes including metabolism, signal transduction, and overall homeostasis. Inadequate regulation of volume can result in cellular dysfunction and pathologies [173, 176].

In summary, through diverse mechanisms involving metabolic support, antioxidant defense, and signaling regulation, glutamine functions as a central metabolic hub that enables cells to adapt to various stress conditions. This section highlights the dynamic regulation of glutamine metabolism under different cellular stresses and its association with stress‐related diseases (Figure 3).

**FIGURE 3:**
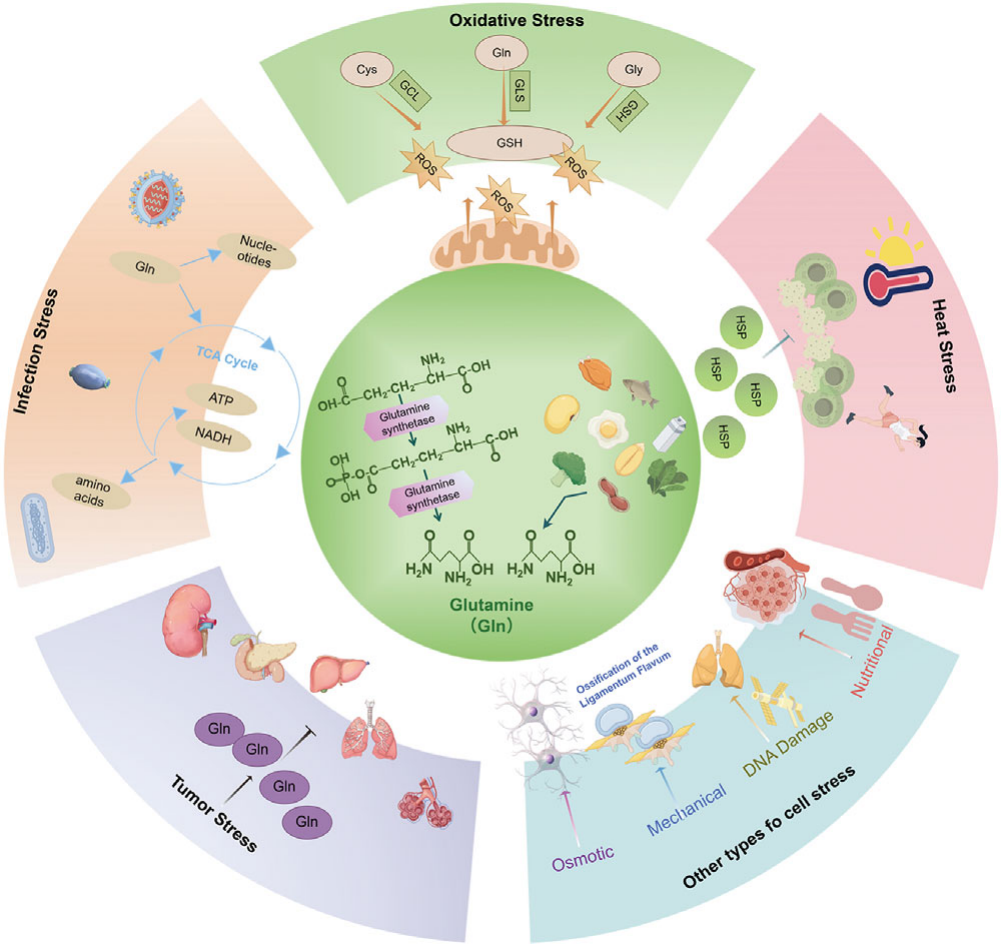
The state of cellular glutamine metabolism under various stress conditions. The body can acquire glutamine in two ways: either by synthesizing it internally or by directly obtaining it from dietary sources. Under different stress conditions, the body will respond differently. Under oxidative stress, glutamine acts as a precursor to glutathione, playing a vital role in its synthesis. This process is essential for protecting cells from damage induced by reactive oxygen species (ROS). Hot environments or strenuous exercise can lead to heat stress in humans. In response to heat stress, glutamine upregulates the levels of heat shock proteins (HSPs), which serve to protect cells and inhibit apoptosis. Nutritional stress can arise from insufficient nutrient intake or tumor presence. In such situations, increased glutamine catabolism provides energy for tumor cell proliferation, promoting tumor growth and inhibiting cell death induced by reactive oxygen species (ROS). Tissue transglutaminase is involved in the osteogenic differentiation of human ligamentum flavum cells in response to mechanical loading. Additionally, mechanical signals from the tumor microenvironment influence cellular mechanics and modify glutamine metabolism, ultimately enhancing the aggressiveness of cancer cells. Under hypertonic conditions, the levels of L‐glutamine increased in incubated human fibroblasts. Conversely, in a low osmolality environment, the release of L‐glutamine also rises. Similarly, after exposure to silica, there is an observed increase in glutamine levels in human alveolar type II epithelial cells. Facing different stimuli, such as viral, bacterial, and fungal infections, can induce metabolic reprogramming in immune cells. Glutamine metabolism plays a crucial role in various types of cancers, like pancreatic ductal adenocarcinoma, kidney cancer, non–small cell lung cancer, and hepatocellular carcinoma.

## 5 Glutamine Metabolism in Cancer

### 5.1 Oncogenic Regulation of Glutamine Metabolism

Amino acids are essential for cancer cell proliferation, particularly under conditions of genotoxic, oxidative, and nutritional stress [177, 178]. They serve not only as building blocks for protein synthesis but also as metabolic substrates for the generation of glucose, lipids, and nucleotides [179]. Several studies have demonstrated that glutamine catabolism is critical for maintaining TCA cycle flux and supporting cancer cell survival [180]. And glutamine provides nitrogen for the biosynthesis of various amino acids [30]. Several studies have demonstrated that both oncogenic activation and tumor‐suppressive alterations in cancer cells can rewire glutamine metabolism. Moreover, cancer cells present increased levels of reactive oxygen species (ROS), which function not only as damaging molecules but also as signaling mediators that promote tumor growth and metastasis [181, 182]. Maintaining high intracellular GSH levels in cancer cells can help eliminate excess ROS and detoxify xenobiotics, thereby preventing oxidative damage. In addition to its metabolic functions, glutamine contributes to cellular adaptation to acidic environments. Following uptake into *E. coli*, glutamine is converted to L‐glutamate by the acid‐activated enzyme glutaminase YbaS, which concurrently releases gaseous ammonia. The generated ammonia neutralizes intracellular protons, leading to an increase in intracellular pH under acidic conditions [183]. Furthermore, they proposed that glutamine plays a significant role in cancer cell growth through its involvement in acid resistance [184]. Collectively, these findings highlight the essential role of glutamine metabolism in tumor regulation and progression (Figure 4).

**FIGURE 4:**
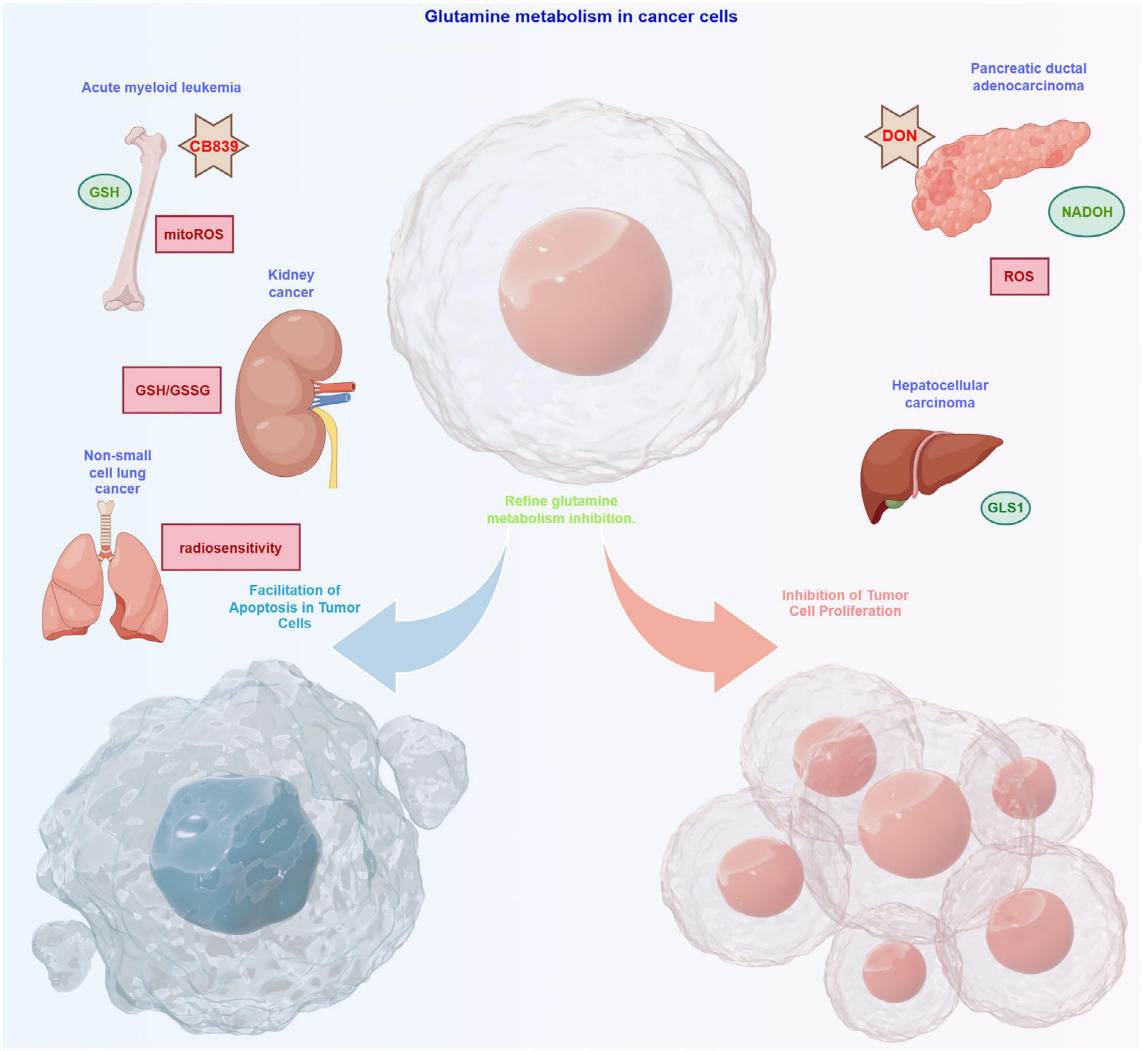
Glutamine metabolism in cancer cells. Glutamine metabolism plays a crucial role in various types of cancers. Inhibiting glutamine metabolism can hinder tumor development by promoting apoptosis in tumor cells and inhibiting tumor cell proliferation. For instance, the glutaminase inhibitor CB‐839 has been shown to disrupt glutamine metabolism in Acute myeloid leukemia, resulting in decreased GSH levels, increased mitoROS, and elevated apoptosis. Knocking down UCP2, the aspartate transporter, reduces glutamine‐derived NADPH levels and increases ROS in PDAC cells, ultimately inhibiting their growth. In kidney cancer, CB‐839 leads to a decreased GSH/GSSG ratio and increased oxidative stress and apoptosis in ccRCCs. Additionally, knockdown of GLS to inhibit glutamine metabolism can disrupt oxidative homeostasis and increase radiosensitivity in NSCLC. Furthermore, GLS1 expression serves as a sensitive and specific biomarker for the pathological diagnosis and prognosis of hepatocellular carcinoma.

### 5.2 Cancer Type‐Specific Dependencies

#### 5.2.1 Acute Myeloid Leukemia

Increasing evidence indicates that glutamine metabolism is involved in the progression of acute myeloid leukemia (AML) [185, 186, 187], and glutamine depletion suppresses mTORC1 signaling and induces apoptosis in AML cells [188]. Glutamine levels control mitochondrial OXPHOS in AML cells [52]. GSH depletion is a common consequence of glutamine metabolism inhibition in AML. Furthermore, this inhibition renders AML cells more susceptible to combination therapies that disrupt oxidative homeostasis [189]. Notably, impaired glutamine metabolism caused by FMS‐like tyrosine kinase 3 (FLT3) inhibitors may result in GSH depletion and an accumulation of mitochondrial reactive oxygen species (mitoROS), ultimately leading to the apoptosis of AML cells [190]. Consistently, treatment of AML cell lines with the glutaminase inhibitor CB‐839 results in decreased GSH levels, increased mitoROS, and elevated apoptosis, indicating that disruption of glutamine metabolism compromises redox homeostasis [191].

#### 5.2.2 Pancreatic Ductal Adenocarcinoma

Increasing evidence suggests that altered glutamine decrease is associated with pancreatic cancer progression [192]. Pancreatic ductal adenocarcinoma (PDAC) cells rely on glutamine metabolism to support proliferation [193]. In PDAC cells, oncogenic KRAS reprograms glutamine metabolism by increasing GOT1 activity while reducing GLUD1 expression, which boosts the NADPH/NADP (+) ratio for better redox stability [194]. Furthermore, GLS is upregulated in human PDAC specimens compared with adjacent normal pancreatic tissues, and glutamine deprivation inhibits the proliferation of PDAC cells more significantly than that of normal pancreatic cells [195]. Additional evidence has shown that knocking down UCP2, the aspartate transporter, reduces glutamine‐derived NADPH levels and increases ROS in PDAC cells, ultimately inhibiting their growth [196]. Therefore, broadly targeting glutamine metabolism could provide a promising therapeutic strategy for PDAC [193]. Recent reports indicate that 6‐diazo‐5‐oxo‐L‐norleucine (DON), a glutamine antagonist that broadly inhibits glutamine metabolism, effectively suppresses tumor growth and metastasis in PDAC [197]. In addition, AS pulvinone O acts as a potent bioactive inhibitor of GOT1 and represents a novel antitumor agent for PDAC therapy [198].

#### 5.2.3 Kidney Cancer

Renal carcinoma cells are highly dependent on glutamine as their energy source, and MYC drives the development of renal cell carcinoma (RCC) in conditional transgenic mouse models through its role in glutamine metabolism; inactivation of MYC can subsequently induce sustained tumor regression [199]. In clear cell renal cell carcinoma cells (ccRCCs), mutations or deletions of the tumor suppressor gene von Hippel‐Lindau (VHL) result in dysregulated hypoxia‐inducible factors (HIFs) and their pro‐oncogenic mediators, including various growth factors and receptors [200, 201]. HIF activation is essential for inducing reductive carboxylation of α‐ketoglutarate in RCC cells, and it also renders VHL‐deficient cells sensitive to glutamine deprivation in vitro [202]. Combined proteomics and metabolomics studies have demonstrated that ccRCCs primarily utilize glutamine to support the GSH‐oxidized antioxidant system for reducing oxidative stress, rather than using it to produce energy and cellular components via the TCA cycle [203]. Miess et al. [87] showed that inhibiting fatty acid metabolism via impaired β‐oxidation forces RCC cells to rely on the glutamine‐glutathione pathway to combat lipid peroxidation and ferroptotic cell death. Another study indicated that inhibiting glutamine metabolism with CB‐839 resulted in a decreased GSH/GSSG ratio, which further increased oxidative stress and apoptosis in ccRCCs.

#### 5.2.4 Non–Small Cell Lung Cancer

Non–small cell lung cancer (NSCLC) relies on glutamine‐derived GSH to maintain oxidative homeostasis to resist radiotherapy [163, 204, 205]. Another study indicated that in NSCLC cells, ROS‐mediated metabolic reprogramming promotes oxidative phosphorylation, leading to cisplatin resistance [206]. In A549 cells, matrix detachment leads to the inhibitory phosphorylation of acetyl‐CoA carboxylase, which not only reduces fatty acid synthesis but also enhances fatty acid oxidation. This inhibition conserves NADPH, making it available to bolster antioxidant defenses against oxidative stress, thereby mitigating cell death that would otherwise occur [207]. An existing study has linked glutamine metabolism to radioresistance in NSCLC [208]. In addition, glutamine metabolism in NSCLC involves various enzymes and transporters, which are regulated at both transcriptional and posttranscriptional levels [209]. Statistical analysis has shown that overexpression of SLC15A is associated with poor prognosis in NSCLC patients, suggesting that it could serve as an important prognostic marker [210]. Furthermore, inhibition of glutamine metabolism through GLS knockdown disrupts redox homeostasis and increases radiosensitivity in NSCLC cells [163, 204]. Collectively, targeting glutamine metabolism may represent a promising therapeutic strategy by disrupting oxidative balance in cancer cells.

#### 5.2.5 Hepatocellular Carcinoma

At present, increasing evidence points to the involvement of metabolic stress in the initiation of primary liver cancers, over 90% of which are hepatocellular carcinoma [211]. Although glutamine is a nonessential amino acid, hepatocellular carcinoma cells depend on it for survival [211]. Soukupova et al. [212] indicated that in hepatocellular carcinoma cells, epithelial–mesenchymal transition involves an increase in GLS1, which transforms glutamine into glutamate, leading to higher levels of TCA intermediates without altering TCA enzyme levels. Similarly, GLS1 overexpression promotes proliferation in hepatocellular carcinoma cells with the involvement of the AKT/glycogen synthase kinase 3 beta/cyclin D1 axis [213]. Furthermore, GLS1 attenuates stemness properties in hepatocellular carcinoma by increasing ROS accumulation and suppressing the Wnt/β‐catenin pathway [214]. GLS1 expression has been proposed as a sensitive and specific biomarker for the pathological diagnosis and prognosis of hepatocellular carcinoma [215].

### 5.3 Metabolic Plasticity and Resistance

Although several inhibitors targeting glutamine metabolism have been developed, the heterogeneity and metabolic plasticity of glutamine metabolism remain major contributors to therapeutic resistance in cancer. Research indicates that cancer cells respond to glutamine starvation by downregulating P5CS, the rate‑limiting enzyme for proline biosynthesis, thus enhancing de novo glutamine production and exposing a previously unrecognized mechanism of metabolic plasticity [216]. Inhibition of glutamine metabolism in tumor cells induces compensatory activation of glycolysis and PD‐L1‐mediated immune evasion, thereby substantially reducing the efficacy of glutamine‐targeted therapies [217]. Moreover, glutamine‐addicted breast cancer cells adapt to chronic glutamine deprivation or GLS inhibition through AMPK‐mediated activation of the serine biosynthesis pathway [218]. Recent studies have revealed that LSD1 transcriptionally regulates GLS2 expression to reprogram glutamine metabolism and promote reductive carboxylation in KRAS‐mutant pancreatic cancer cells, thereby contributing to subtype‐specific chemoresistance [219]. Furthermore, glutamine deprivation or metabolic inhibition induces ATF4 upregulation through stress‐response signaling pathways in cancer cells. Collectively, these findings indicate that targeting glutamine metabolism alone is unlikely to achieve durable antitumor efficacy. Therefore, combination therapeutic strategies or interventions targeting key regulatory nodes of metabolic adaptation may be required to overcome resistance and improve clinical outcomes.

## 6 Glutamine in Immunometabolism

Glutamine serves as a central metabolic substrate for immune cells, supporting energy production and regulating immune cell activation. Upon activation, immune cells undergo extensive metabolic reprogramming, including alterations in glutamine metabolism, to support rapid proliferation and effector functions. Glutamine uptake mediated by SLC1A5 promotes naïve T‐cell activation and modulates T‐cell immune responses [220]. In addition, glutamine can reprogram the metabolic pathways of natural killer cells by maintaining cMyc expression, thereby regulating their effector functions, and this effect is an important part of its influence on the lymphocyte metabolic network [221]. It also plays a crucial role in the metabolism of B lymphocytes. Inhibition of GLS expression reduces immunoglobulin antibody production, while GLS blockade suppresses mTOR activation and dampens IL‐10 secretion [222]. Tumor‐associated macrophages regulate their activation status, synthesis of inflammatory cytokines, and peroxisome proliferation through modulating glutamine metabolism [71]. Recent studies have demonstrated that targeting glutamine metabolism with glutamine antagonists to reprogram macrophage immunometabolism enhances therapeutic efficacy against colorectal cancer in mouse models [223]. Collectively, a comprehensive understanding of how glutamine metabolism is regulated across distinct immune cell subsets and disease contexts will facilitate the development of more precise and effective immunotherapeutic strategies.

## 7 Therapeutic Strategies Targeting Glutamine Metabolism

Increasing evidence has highlighted multiple therapeutic strategies targeting glutamine metabolism, including dietary modulation and pharmacological intervention. Dietary manipulation of glutamine availability may induce metabolic and epigenetic reprogramming in tumor cells, thereby enhancing therapeutic sensitivity. In parallel, small‐molecule inhibitors targeting key components of the glutamine metabolic network, including GLS inhibitors, glutamine transport inhibitors, and broad‐spectrum glutamine metabolism inhibitors, have emerged as promising candidates in preclinical oncology studies, with several compounds progressing toward clinical evaluation (Table 2).

**TABLE 2:** Clinical trials landscape of glutamine‐targeted therapies.

Mechanism	Inhibitors	Indication	Clinical stage	Major limitations	Ref.
Glutaminase (GLS1) inhibitors	BPTES	Hepatocellular carcinoma; triple‐negative breast cancer	Preclinical Stage	Poor solubility hinders its clinical translation	[237, 238, 239]
	CB‐839 (Telaglenastat)	Non–small cell lung cancer; triple‐negative breast cancer; renal cell carcinoma; colorectal; myelodysplastic syndrome; leukemia	Clinical Phase I/II NCT03831932 NCT03057600 NCT03428217 NCT03798678 NCT02071927	Limited efficacy of monotherapy	[240, 241, 242, 243, 244]
Broad‐spectrum inhibitors of glutamine metabolism	JHU‐083	Major depressive disorder; malignant glioma; medulloblastoma	Preclinical Stage	Demonstrates low tumor selectivity	[231, 245, 246, 247]
	DRP‐104	Pancreatic ductal adenocarcinoma; non–small cell lung cancer; KEAP1 mutant lung cancer	Clinical Phase I/II NCT07249372	The efficacy of broad‐spectrum monotherapy is sub‐optimal	[193, 230]
Glutamine transporter inhibitors	GPNA	Gastric cancer; colorectal	Preclinical Study	GPNA is a low‐affinity competitive system L transporter inhibitor	[248, 249, 250]
	V‐9302	TNBC; uveal melanoma; pancreatic cancer; mesothelioma; colorectal cancer	Preclinical Study	Its poor solubility in aqueous solutions	[251, 252, 253, 254, 255, 256]

### 7.1 Dietary and Pharmacological Modulation

Recent studies have demonstrated that glutamine supplementation can restore glutamine levels in skeletal muscle, decreasing oxidative stress and inflammation [224]. Parenteral administration of glutamine has also been reported to provide clinical benefits in patients undergoing surgery, radiotherapy, bone marrow transplantation, or severe injury [61]. Conversely, metabolic restriction of glutamine availability represents another potential therapeutic strategy. Simultaneous deprivation of glucose and glutamine effectively suppresses tumor growth in orthotopic pancreatic cancer models [225]. In addition, inhibition of branched‐chain amino acid (BCAA) and glutamine metabolism further restricts hepatocellular carcinoma progression in vivo [226].

### 7.2 Small Molecule Inhibitors in Preclinical and Clinical Development

Small‐molecule inhibitors targeting glutamine metabolism currently under preclinical and clinical investigation are summarized in Table 2. Among these approaches, glutaminase targeting represents one of the most extensively studied strategies. Glutaminase inhibitors can be broadly classified into two major categories: allosteric inhibitors and glutamine analogs [227]. CB‐839 is a clinically evaluated glutaminase inhibitor and a representative example of allosteric GLS inhibition. It was developed through rational optimization of the lead compound bis‐2‐(5‐phenylacetamido‐1,3,4‐thiadiazol‐2‐yl) ethyl sulfide (BPTES), resulting in improved pharmacological properties and enhanced inhibitory activity against GLS [228]. Currently, it is the dominant compound in this category and is in the second phase of clinical trials [229]. Glutamine analogs share structural similarity with the substrate but typically incorporate alternative functional groups in place of the amide moiety, and they bind directly to the catalytic active site. 6‐Diazo‐5‐oxo‐L‐norleucine (DON) is a glutamine antagonist that potently and irreversibly inhibits multiple enzymes involved in glutamine metabolism. Its prodrug, DRP‐104, has demonstrated therapeutic efficacy in preclinical models of lymphoma, colorectal cancer, lung cancer, and pancreatic cancer, and is currently under clinical investigation [193, 230, 231]. Collectively, these findings highlight the therapeutic potential of targeting cancer metabolic reprogramming.

V9302 is a selective small‐molecule antagonist targeting the amino acid transporter SLC1A5 [232]. Previous studies have demonstrated that V9302 exhibits substantial antitumor activity and may provide advantages over certain glutamine metabolism inhibitors, including CB‐839 [233]. Pharmacological inhibition of SLC1A5 by V9302 reduces tumor cell proliferation and increases apoptosis [232]. Other studies have also demonstrated that V9302 promotes cellular senescence and inhibits tumor growth, invasion, and migration [234]. V‐9302 exerts antitumor effects by reducing GSH levels and mTOR activity, as well as inducing autophagy, oxidative stress, and immune responses [235, 236]. Despite demonstrating favorable antitumor efficacy in several cancer models, V‐9302 exhibits poor water solubility and potential off‐target effects. Additionally, its interference with glutamine metabolism leads to a compensatory increase in glucose metabolism in tumor cells.

### 7.3 Combination Therapies and Resistance Mechanisms

Treatment resistance stands as a primary obstacle in oncology, especially in the context of cancer heterogeneity. The intricate cellular architecture within tumors renders single targeted agents or chemotherapies prone to selective enrichment, leading to the outgrowth of resistant clones. Nevertheless, while these mutant cells are profoundly distinct at the genomic level, they demonstrate remarkable consistency in their metabolic wiring. Compared with their nonmalignant counterparts, malignant cells are significantly more reliant on glucose, glutamine, and alternative substrates. Given that aberrant Gln metabolism is intricately coupled with the acquisition of tumor drug resistance, and with studies demonstrating that tumor proliferation is highly dependent on glutamine [257], addressing metabolic heterogeneity and plasticity frequently necessitates combination therapies tailored to the patient's pathological status.

To date, targeting glutamine metabolism alongside standard therapies has shown great therapeutic promise. For instance, combining glutamine metabolism inhibitors with a KD shows strong antitumor effects in pancreatic cancer models [258]. In TNBC, monotherapy with the glutaminase inhibitor CB‐839 or its combination with paclitaxel significantly inhibits tumor growth [240]. Similarly, CB‐839 in combination with erlotinib has demonstrated clinical utility for the treatment of NSCLC [241]. Beyond lung cancer, the inhibition of glutamine uptake by GPNA remarkably potentiates the efficacy of cetuximab against gastric cancer proliferation in vitro and in vivo [248]. Furthermore, combining glutamine metabolism inhibition with immune checkpoint blockade for the treatment of TNBC [259]. However, the metabolic cross‐talk within the immune microenvironment can be double‐edged. When combined with immunotherapy, CB‐839 has been demonstrated to inhibit the clonal expansion and activation of CD8 T cells in NSCLC, thereby compromising the therapeutic efficacy of anti‐PD‐1 treatment [260]. Otherwise, DRP‐104, administered either as a monotherapy or in combination with immune checkpoint blockade therapy, has the potential to improve clinical cure rates in patients with cancer [261]. Combining a DON prodrug with gefitinib reverses PD‐L1 upregulation, thereby alleviating T‐cell immunosuppression and enhancing therapeutic outcomes for bladder cancer [262]. Although multitarget regulation of glutamine metabolism is essential for cancer therapy, its application is limited by uncontrolled drug biodistribution in vivo. Therefore, precise co‐delivery strategies are urgently needed. To address this, a novel nanomodulator achieves ratio‐precise co‐delivery of metabolic drugs in vivo, synergistically disrupting glutamine uptake and utilization in PDAC [263]. Similarly, an injectable short‐peptide gel scaffold co‐loaded with the glutaminase inhibitor CB‐839 and copper peptide nanoparticles was developed, integrating metabolic inhibition with chemodynamic therapy [264]. Furthermore, an SLC1A5‐targeted nanoagonist suppressed glutamine uptake, thereby reversing chemoresistance in cervical carcinoma by downregulating cisplatin efflux and DNA repair [265]. Collectively, these findings demonstrate that precise co‐delivery systems targeting glutamine pathways, whether applied as monotherapies or combined with multimodal regimens, represent a promising approach for diverse malignancies.

## 8 Methods and Emerging Technologies

Given that glutamine metabolism supports tumor growth and metabolic adaptation, glutamine‐targeted tracers hold significant promise for broad‐spectrum tumor visualization. To date, several glutamine‐backbone positron emission tomography (PET) tracers have successfully entered clinical evaluation, offering important insights for imaging gliomas, breast cancer, and various other malignancies [266, 267, 268]. Among these, 5–^11^C‐(2*S*)‐glutamine and ^18^F‐(2*S*,4*R*)4‐fluoroglutamine are pivotal for probing in vivo tumor glutamine metabolism [269]. Specifically, 5–^11^C‐(2*S*)‐glutamine is transported into cancer cells and converted by glutaminase into glutamate, which subsequently contributes to TCA cycle metabolism. Beyond cell‐intrinsic utilization, spatial metabolic communication also shapes the tumor microenvironment. Li et al. [270] developed an approach that comprehensively traces the in vivo fate of ^13^C‐nutrients to characterize spatial metabolic communications in situ, revealing that glucose‐derived glutamine released from the lung serves as a potential source for tumor glutamate synthesis. In light of this systemic metabolic cross‐talk, ^18^F‐fluoroglutamine PET imaging represents a promising tool for evaluating in vivo glutamine metabolism in cancer patients and may facilitate the development of glutamine‐targeted therapies.

Single‐cell metabolomics (SCM) directly reveals cellular metabolic heterogeneity. The goal of SCM is to provide unbiased quantitative characterization of metabolites in single cells, thereby capturing functional diversity [271]. Using SCM, researchers identified two gefitinib‐responsive subpopulations in NSCLC cells, among which a 14.4% subpopulation exhibited relatively stable glutathione metabolism, consistent with a drug‐resistant metabolic phenotype [272]. SCM has also enabled discrimination of cancer subtypes, characterization of glucose‐deprivation responses in MCF7 cells, and identification of metabolic regulation associated with stem‐cell states [273]. Therefore, SCM provides a powerful platform for high‐coverage and high‐sensitivity analysis of cellular metabolic heterogeneity.

Genome‐wide CRISPR–Cas9 screening has evolved from a discovery approach into a powerful functional genomics platform for identifying therapeutic vulnerabilities in cancer. Whole‐genome CRISPR screening revealed that glutamine inhibition induces rapid polyunsaturated fatty acid (PUFA) accumulation through autophagy‐mediated nutrient sensing, thereby identifying mechanisms underlying glutamine sensitivity and resistance [274]. Similarly, CRISPR interference screening identified TARBP1 as a critical regulator of glutamine dependency in cancer cells [275]. Furthermore, a CRISPR‐based SLC transporter screen identified SLC38A1 as a glutamine transporter selectively required for Th1 cell function [276]. Collectively, these findings demonstrate the utility of CRISPR screening in uncovering glutamine‐associated metabolic vulnerabilities and informing rational combination therapeutic strategies.

## 9 Conclusion and Future Perspectives

Given the fundamental importance of glutamine metabolism in maintaining physiological homeostasis and the widespread exposure of organisms to diverse stressors, this review provides an updated overview of glutamine metabolic regulation under various stress conditions and its involvement in stress‐associated diseases. We summarize how glutamine metabolism contributes to cellular adaptation to oxidative, heat, nutritional, mechanical, DNA damage, and osmotic stresses through regulation of energy production, redox balance, and biosynthetic capacity. As a central metabolic hub, glutamine supports cellular survival by maintaining bioenergetic homeostasis, modulating oxidative stress responses, and supplying carbon and nitrogen for macromolecule synthesis. Many major disorders, including central nervous system diseases and cancers, are increasingly recognized as disorders associated with redox imbalance. The primary defense mechanism against oxidative stress in the body is GSH. As a precursor to GSH, glutamine plays an essential role in the management of redox diseases. Under oxidative stress, increased demand for GSH drives enhanced glutamine uptake and metabolism, thereby facilitating ROS detoxification and limiting oxidative damage. In cancer cells and hypoxic tissues, increased glycolytic activity often results in reduced mitochondrial glucose oxidation and depletion of TCA cycle intermediates. Generating α‐KG, glutamine sustains TCA cycle operation to produce ATP and NADPH, supporting cell survival during energy deprivation or when oxidative phosphorylation is impaired. When proliferation or repair remains necessary under stress (such as during tissue damage or immune responses), glutamine provides essential carbon and nitrogen sources for the synthesis of nucleotides, proteins, and lipids. In neurodegenerative diseases, impaired glutamine–glutamate cycling in astrocytes and neurons leads to excitotoxicity, neuroinflammation, and neuronal death. Recent advances in metabolomics and cell biology have deepened our understanding of the networks regulating glutamine metabolism, including key enzymes and signaling pathways [277]. Targeting these regulatory nodes has emerged as a potential strategy for treating diseases caused by glutamine metabolic dysregulation, with several small‐molecule inhibitors and dietary interventions currently progressing through preclinical or clinical evaluation [278].

In the future, to translate these fundamental discoveries into tangible clinical benefits, well‐designed, large‐scale clinical trials are required to systematically evaluate the efficacy and safety of glutamine metabolism‐targeted therapies across diverse patient populations. Patients should be stratified by disease subtype, progression stage, genetic background, and other relevant factors to identify optimal responders and minimize off‐target effects. High‐throughput screening can be employed to discover novel small‐molecule inhibitors targeting key enzymes in glutamine metabolism, thereby facilitating the development of new targeted drugs and biomarkers. Despite significant advances in understanding glutamine metabolism in the context of stress and disease, several key knowledge gaps remain to be addressed. First, the precise crosstalk between glutamine metabolism and other nutrient metabolic pathways, such as glycolysis and fatty acid metabolism, specifically under pathological conditions has not been fully elucidated. Deciphering the synergistic or antagonistic interactions among these pathways will provide novel insights into the metabolic reprogramming of diseased cells. Second, dissecting the context‐specific regulatory networks of glutamine metabolism across distinct stress stimuli, together with its cell‐type and tissue‐specific characteristics, is critical for the rational design of more effective targeted therapeutic strategies. Finally, future studies should further clarify the clinical applications of glutamine modulation under different stress conditions and pathological settings, while exploring the potential synergistic effects of combining glutamine‐targeted therapies with other therapeutic agents. Meanwhile, long‐term studies are needed to evaluate the safety, efficacy, and sustainability of glutamine‐directed interventions, with the ultimate goal of translating stress‐specific metabolic adaptations into precision therapeutic strategies that improve clinical outcomes in patients with diverse stress‐related diseases.

## Author Contributions

Yi Ding and Fan Tong wrote the manuscript and drew the figures. Jing Zeng contributed to the revision of the manuscript. Chun‐Ping Cui evaluated and reviewed the manuscript structure and ideas. All authors have read and approved the final manuscript.

## Ethics Statement

No ethical approval was required for this study.

## Conflicts of Interest

The authors declare no conflicts of interest.

## Data Availability

Data availability is not applicable to this article as no new data were created or analyzed in this study.
